# Wax ester production in nitrogen-rich conditions by metabolically engineered *Acinetobacter baylyi* ADP1

**DOI:** 10.1016/j.mec.2020.e00128

**Published:** 2020-04-25

**Authors:** Jin Luo, Elena Efimova, Pauli Losoi, Ville Santala, Suvi Santala

**Affiliations:** Faculty of Engineering and Natural Sciences, Hervanta Campus, Tampere University, Korkeakoulunkatu 8, Tampere, 33720, Finland

**Keywords:** Storage lipids, Wax ester, Metabolic engineering, Protein-rich substrate, *Acinetobacter baylyi* ADP1, WEs, wax esters, TCA, tricarboxylic acid, FBA, flux balance analysis

## Abstract

Metabolic engineering can be used as a powerful tool to redirect cell resources towards product synthesis, also in conditions that are not optimal for the production. An example of synthesis strongly dependent on external conditions is the production of storage lipids, which typically requires a high carbon/nitrogen ratio. This requirement also limits the use of abundant nitrogen-rich materials, such as industrial protein by-products, as substrates for lipid production. *Acinetobacter baylyi* ADP1 is known for its ability to produce industrially interesting storage lipids, namely wax esters (WEs). Here, we engineered *A. baylyi* ADP1 by deleting the gene *aceA* encoding for isocitrate lyase and overexpressing fatty acyl-CoA reductase Acr1 in the wax ester production pathway to allow redirection of carbon towards WEs. This strategy led to 3-fold improvement in yield (0.075 ​g/g glucose) and 3.15-fold improvement in titer (1.82 ​g/L) and productivity (0.038 ​g/L/h) by a simple one-stage batch cultivation with glucose as carbon source. The engineered strain accumulated up to 27% WEs of cell dry weight. The titer and cellular WE content are the highest reported to date among microbes. We further showed that the engineering strategy alleviated the inherent requirement for high carbon/nitrogen ratio and demonstrated the production of wax esters using nitrogen-rich substrates including casamino acids, yeast extract, and baker’s yeast hydrolysate, which support biomass production but not WE production in wild-type cells. The study demonstrates the power of metabolic engineering in overcoming natural limitations in the production of storage lipids.

## Highlights

• Efficient wax ester (WE) accumulation with low carbon/nitrogen ratio was demonstrated
• Deletion of *aceA* and overexpression of *acr1* enhanced WE production in *Acinetobacter baylyi* ADP1
• The highest WE titer, productivity, and cellular WE content reported to date was achieved
• Engineered strain produced WEs directly from nitrogen-rich substrates
• Metabolic engineering alleviated the native restrictions behind WE synthesis

## 1 Introduction

Metabolic engineering provides a powerful tool for improving the production of industrially relevant products using microbial cells as factories (Pfleger et al., 2015). One potential approach is to optimize the distribution of carbon and cellular resources between biomass and product synthesis, which is a common challenge in microbial production. Microbial storage lipid production represents a typical example of a synthesis route that strongly competes with biomass production. To induce lipid accumulation, excess of carbon along with the limitation of some key nutrients, typically nitrogen, is required (Breuer et al., 2012; Garay et al., 2014; Wältermann and Steinbüchel, 2005). Therefore, the production process is typically divided into two phases, where biomass is accumulated in the first phase and the lipid accumulation is induced in the second phase by limiting nitrogen availability while sustaining high carbon substrate concentration. However, such approach can result in low overall productivity. In addition, nitrogen limitation may not be suitable for cells (over)expressing non-native activities and pathways. One strategy to manipulate the distribution of carbon and energy between biomass and product synthesis is to directly engineer the central carbon metabolism. However, the deletion of key genes in central metabolism may harm cell functions and strongly reduce growth, which can negatively affect production. Thus, in previous studies, various strategies for dynamic control of enzyme levels in central metabolism have been introduced. For example, Soma et al. increased the availability of acetyl-CoA from the tricarboxylic acid (TCA) cycle for isopropanol production by conditionally switching off the expression of citrate synthase (Soma et al., 2014). In another example, Doong et al. improved the production of glucaric acid by dynamically down-regulating an essential enzyme in the competing glycolytic pathway based on quorum sensing and up-regulating a downstream enzyme upon the production of its intermediate (Doong et al., 2018). More recently, Santala et al. established autonomous downregulation of the glyoxylate shunt based on the gradual oxidation of the inducer arabinose, enabling a shift from biomass formation to lipid accumulation (Santala et al., 2018).

Wax esters (WEs) are an example of high-value storage lipids, which could be used in a broad range of applications, including cosmetics, lubricants, pharmaceuticals, printing, food industries, etc. (Sánchez et al., 2016; Singh et al., 2007). *Acinetobacter* sp. is the best-known microorganism for producing long-chain WEs as energy and storage compounds (Fixter et al., 1986; Rontani, 2010). In addition, *Acinetobacter baylyi* ADP1, is an ideal cellular platform for synthetic biology and metabolic engineering due to its genetic tractability (de Berardinis et al., 2008; Metzgar, 2004; Murin et al., 2012; Suárez et al., 2017; Tumen-Velasquez et al., 2018), thus enabling it to be a suitable host to study storage lipid production (Santala et al., 2014). Besides WEs, *A. baylyi* ADP1 has also been engineered to produce various native and non-native oleochemicals, such as alkanes, alkenes, and triacylglycerols (Lehtinen et al., 2018b; Luo et al., 2019; Salmela et al., 2019; Santala et al., 2011b).

**Fig. 1:**
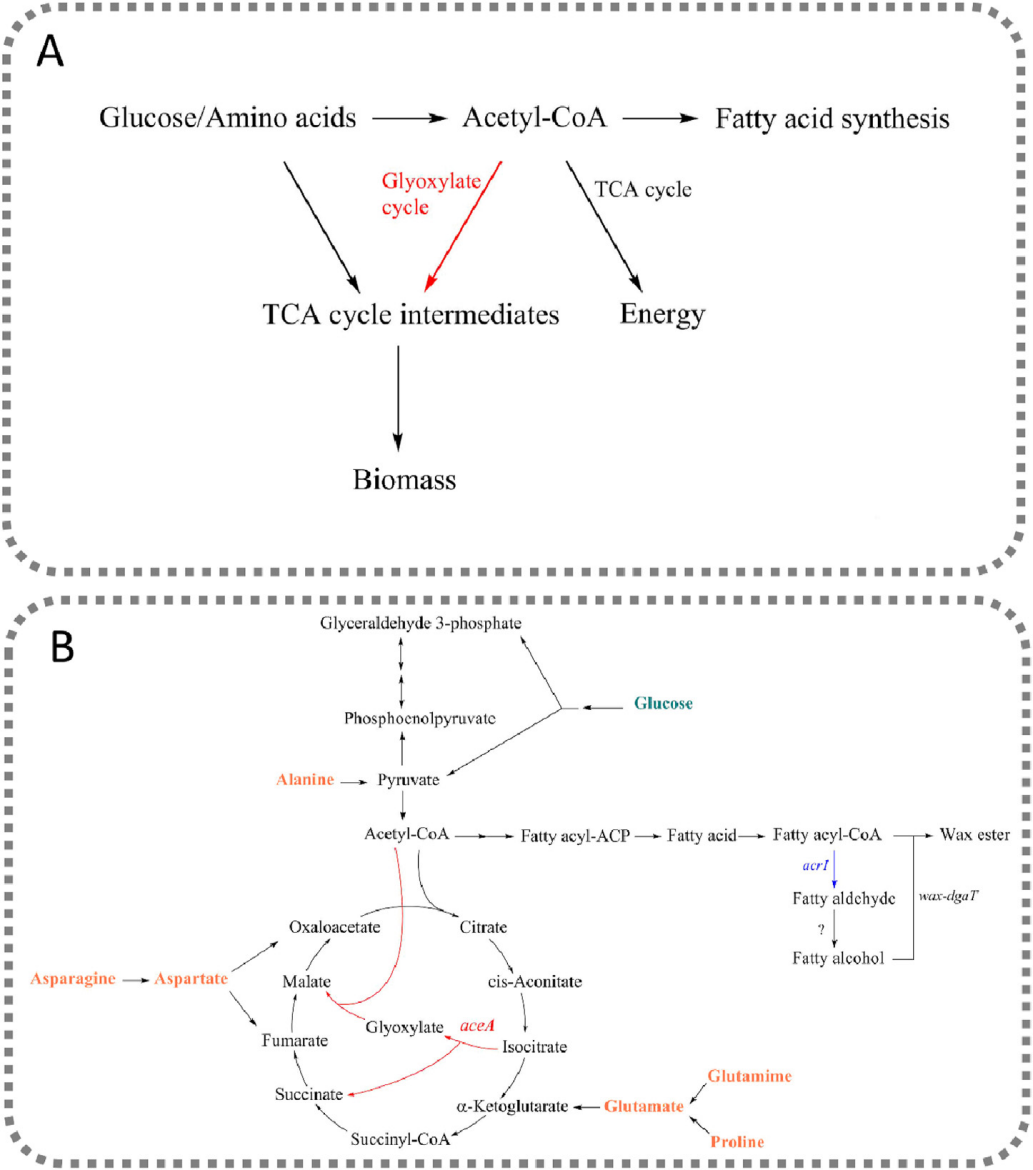
(A) Removal of the glyoxylate shunt hypothetically increases the availability of the central metabolite, acetyl-CoA, for fatty acid synthesis and energy generation. (B) The simplified metabolic pathway for the synthesis of wax esters from glucose and amino acids by *A. baylyi* ADP1. In the engineered strain, the gene *aceA* encoding for the isocitrate lyase, was deleted, blocking the glyoxylate shunt. The gene *acr1*, encoding for the fatty acyl-CoA reductase, was overexpressed to facilitate wax ester production. In the final step of wax ester synthesis, fatty alcohol is esterified with fatty acyl-CoA by wax ester synthase (wax-dgaT).

The fatty acid synthesis pathway provides the precursors fatty acyl-CoAs (coenzyme A) for the synthesis of WEs (Janβen and Steinbüchel, 2014). The pathway produces fatty acyl-ACPs (acyl carrier protein) as products by iterative enzymatic reaction cycles, starting with the central metabolic intermediate, acetyl-CoA. In *Acinetobacter* sp., the resulting fatty acyl-ACPs are further converted to fatty acyl-CoAs, followed by three enzymatic reactions involved in WE synthesis: (1) reduction of fatty acyl-CoAs to fatty aldehydes by the fatty acyl-CoA reductase Acr1 (Reiser and Somerville, 1997), (2) reduction of the fatty aldehydes to fatty alcohols by an uncharacterized enzyme and (3) esterification between the fatty alcohols and the fatty acyl-CoAs by a bifunctional wax ester synthase/diacylglycerol acyltransferase (WS/DGAT) (Kalscheuer and Steinbüchel, 2003). The glyoxylate shunt, which widely exists in bacteria, hypothetically competes with the fatty acid synthesis pathway for acetyl-CoA (Fig. 1A). Unlike the complete TCA cycle, the glyoxylate shunt bypasses two oxidative steps that release two molecules of carbon dioxide (Kornberg, 1966). Thus, one important role of the glyoxylate shunt is to replenish the intermediates in the TCA cycle that are withdrawn for growth, using two molecules of acetyl-CoA as substrates (Kornberg, 1966). The glyoxylate shunt is essential for cells growing on acetate or other non-glycolytic carbon sources, such as fatty acids, that are directly catabolized into acetyl-CoA. However, when glucose or amino acids are available, the glyoxylate shunt is not essential since TCA cycle intermediates can be supplied by alternative ways. In these cases, blocking the glyoxylate shunt can be hypothesized to enhance lipid production by increasing the flow of the available acetyl-CoA to fatty acid synthesis pathway and energy generation without affecting cell growth (Fig. 1A).

Renewable plant-derived biomass and algal biomass have been emerging as alternatives to fossil fuels for the production of fuels and chemicals (Peralta-Yahya et al., 2012; Pfleger et al., 2015; Ragauskas, 2006; Steen et al., 2010). However, this scenario is accompanied with an accumulation of large amounts of nitrogen-rich by-products (Li et al., 2018). It was estimated that 100 ​Mt/year of protein could be generated if 10% of transportation fuels were produced from biomass-derived sources (Tuck et al., 2012). In addition, considerable amounts of protein-rich wastes are produced from the brewery and food industries (Ferreira et al., 2010; Tuck et al., 2012). It is of great importance to exploit the value of these waste streams to develop a circular economy. Thus, strategies have been brought out for the valorization of protein wastes, of which microbial production of chemicals directly from protein wastes is advantageous, as compared to other processes that require costly amino acid isolation steps (Li et al., 2018). Although the engineering strategies for the bioproduction of chemicals from protein sources have been described (Huo et al., 2011), it is still challenging to efficiently produce oleochemicals, such as storage lipids, from nitrogen-rich protein wastes.

For ADP1, protein-rich substrates provide an excellent carbon and nutrient source for growth, i.e. biomass production. However, due to the high nitrogen content, they are not suitable for the production of WEs. Here, we employ a metabolic engineering strategy aiming at efficient lipid accumulation, even from nitrogen-rich carbon sources, thus bypassing the need for a high carbon/nitrogen ratio. We engineered *A. baylyi* ADP1 by deleting the gene *aceA*, which is essential for the glyoxylate shunt, from the genome and overexpressing the gene *acr1*, which encodes for the fatty acyl-CoA reductase (Fig. 1B). We first evaluated the production of WEs by the engineered strain using glucose as a carbon source. Prior to the evaluation of WE production from nitrogen-rich substrates, we examined the utilization of 20 amino acids as sole carbon source by the modified strains and explored the effect of the modifications on cell growth and lipid synthesis. We finally cultivated the engineered strain with different nitrogen-rich substrates (casamino acids, yeast extract, and baker’s yeast hydrolysate) for WE production. We demonstrate that our approach significantly improved the production of WEs from both glucose and nitrogen-rich substrates.

## 2 Materials and methods

### 2.1 Strains and media

*Acinetobacter baylyi* ADP1 (DSM 24193, DSMZ, Germany) was used in the study for WE production. *E. coli* XL1-Blue (Stratagene, USA) was used as a host in plasmid constructions and amplifications. All strains used in the study are listed in Table 1.

**Table 1:** Bacterial strains used in the study.

Name	Genotype	Description	Source/reference
ADP1 WT	Wild type *A. baylyi* ADP1	Control strain for WE production study.	DSM 24193, DSMZ
ADP1 Δ*aceA*	*A. baylyi* ADP1 Δ*aceA*::*kan*^*r*^	Strain with *aceA* deletion for WE production study.	This study
ADP1 Δ*acr1*	*A. baylyi* ADP1 Δ*acr1*::*kan*^*r*^	Background strain with *acr1* deletion for constructing W1.	de Berardinis et al. (2008)
ADP1 Acr1	*A. baylyi* ADP1 Δ*acr1*::*cm*^*r*^*,* Δpp::P_T5_-*acr1*-*spec*^*r*^	Strain with *acr1* overexpression at the prophage region of ADP1 genome for WE production study.	This study
W1	*A. baylyi* ADP1 Δ*acr1*::*kan*^*r*^, Δ*aceA*::P_T5_-*acr1*-*spec*^*r*^	Strain with *aceA* deletion and *acr1* overexpression for WE production study.	This study
ADP1+iluxAB	*A. baylyi* ADP1 Δ*poxB*::P_T5_-*luxAB*,*cm*^*r*^	Control strain with *luxAB* expression for the study of fatty aldehyde production and amino acid utilization.	Santala et al. (2011a)
ADP1 Δ*aceA* ​+ ​iluxAB	*A. baylyi* ADP1 Δ*aceA*::*kan*^*r*^, Δ*poxB*::P_T5_-*luxAB*,*cm*^*r*^	Strain with *aceA* deletion and *luxAB* expression for the study of fatty aldehyde production.	This study
ADP1 Acr1+iluxAB	*A. baylyi* ADP1 Δpp::P_T5_-*acr1*-*spec*^*r*^, *ΔpoxB,metY,acr1*:: P_T5_-*luxAB*-*kan*^*r*^	Strain with *acr1* overexpression at the prophage region of ADP1 and *luxAB* expression for the study of fatty aldehyde production.	This study
W1+iluxAB	*A. baylyi* ADP1 Δ*acr1*::*kan*^*r*^, Δ*aceA*::P_T5_-*acr1*-*spec*^*r*^, Δ*poxB*::P_T5_-*luxAB*,*cm*^*r*^	Strain with *aceA* deletion, *acr1* overexpression and *luxAB* expression for the study of fatty aldehyde production.	This study
W2+iluxAB	*A. baylyi* ADP1 Δ*aceA*::P_T5_-*acr1*-*kan*^*r*^, *ΔpoxB,metY,acr1*:: P_T5_-*luxAB*-*spec*^*r*^	Control strain with *luxAB* expression for the study of the effect of the mutation in the *acr1* of W1 on fatty aldehyde production.	This study
ADP1 FAR-neg.+iluxAB	*ΔpoxB,metY,acr1*:: P_T5_-*luxAB*-*kan*^*r*^	Negative control strain with *luxAB* expression and without fatty acyl-CoA reductase for the study of fatty aldehyde production.	Lehtinen et al. (2018a)

Modified LB medium (10 ​g/L tryptone, 5 ​g/L yeast extract, 1 ​g/L NaCl) supplemented with 1% glucose was used to grow *E. coli* and *A. baylyi* ADP1 for strain constructions. Cultivations for all the experiments were carried out using modified minimal salts medium MA/9 (Na_2_HPO_4_ 4.40 ​g/L, KH_2_PO_4_ 3.40 ​g/L, NH_4_Cl 1.00 ​g/L, nitrilotriacetic acid 0.008 ​g/L, NaCl 1.00 ​g/L, MgSO_4_ 240.70 ​mg/L, CaCl_2_ 11.10 ​mg/L, FeCl_3_ 0.50 ​mg/L) supplemented with different types of carbon sources as appropriate. For the characterization of amino acid utilization and fatty aldehyde production from amino acids, 20 ​L-amino acids (alanine, arginine, asparagine, aspartic acid, cysteine, glutamic acid, glutamine, glycine, histidine, isoleucine, leucine, lysine, methionine, phenylalanine, proline, serine, threonine, tryptophan, tyrosine, valine, all from Sigma) were added as sole carbon source respectively, with a final concentration of 15 ​mM (except for tyrosine for which the concentration was made to 5 ​mM due to its low solubility). For the characterization of fatty aldehydes and WE production from glucose, 200 ​mM glucose and 2 ​g/L casamino acids were added. For WE production from nitrogen-rich substrates, casamino acids (10 ​g/L and 20 ​g/L), yeast extract (20 ​g/L), and 50% of baker’s yeast hydrolysate (the baker’s yeast was purchased from the local market) were added, respectively. The pretreatment of the baker’s yeast was performed using the method described by Huo et al. (Huo et al., 2011), with some modifications. Briefly, 126 ​g/L biomass was mixed with water and heated at 80–85 ​°C for 18 ​min, after which the biomass was digested with 1–2 ​g/L protease (from *Bacillus* sp., Sigma) and incubated at 60 ​°C overnight. The digestion product was centrifuged, and the supernatant was collected and further filtered for use as a substrate. Corresponding antibiotics were added in the media when necessary (with a concentration of 50 ​mg/L for spectinomycin, 50 ​mg/L for kanamycin, and 25 ​mg/L for chloramphenicol).

### 2.2 Genetic manipulation

The molecular cloning was performed using established methods. The reagents for the molecular work, including PCR, digestion, and ligation, were provided by Thermo Scientific (USA) and used according to the provider’s instruction. Transformation and the homologous recombination-based genome editing of *A. baylyi* ADP1 were carried out as described previously (Santala et al., 2011b). All the primers used in the study are listed in Table S3.

The strain ADP1 Δ*acr1* (ΔACIAD3383) with *acr1* deletion was a kind gift from Dr. Veronique de Berardinis (Genoscope, France). The strain ADP1+iluxAB was previously constructed in our laboratory (Santala et al., 2011a); in the strain, the gene *poxB* (ACIAD3381) is replaced with the genes *luxAB* under control of a T5 promoter. The strain ADP1 FAR-neg.+iluxAB was described previously (Lehtinen et al., 2018a), with three adjacent genes *poxB* (ACIAD3381), metY (ACIAD3382) and *acr1* (ACIAD3383) being replaced with the gene *luxAB*. The genes *poxB* and *metY* are neutral targets for deletion; their deletions have been shown not to have a negative effect on growth and WE production of ADP1 (Santala et al., 2011b).

To construct the strain ADP1 Δ*aceA*, the cassette P_T5_-*kan*^r^ was cloned from previously contructed plasmid P_T5_-*kan*^r^/pAK400c (Santala et al., 2011b) to the plasmid pUC57(Δ*aceA*) containing the sequences flanking the gene *aceA* in the ADP1 genome (purchased from GenScript, USA) by digestion and ligation with the restriction enzymes AvrII and MfeI. The resulting plasmid pUC57(Δ*aceA*)- P_T5_-*kan*^r^ was used to transform ADP1 WT to obtain the strain ADP1 Δ*aceA*. The construction of the strain W1 was carried out as follows. First, the gene *acr1* (amplified from the genome of *A. baylyi* ADP1 using the primers tl17 and sa16) was cloned to a previously described chromosomal integration cassette (Santala et al., 2011a) under control of a strong constitutive promoter T5. The original chloramphenicol resistance marker in the cassette was replaced with a spectinomycin resistance marker (amplified from the plasmid pIM1463 (Murin et al., 2012) with the primers tl45 and tl46). The resulting cassette P_T5_-*acr1*-*spec*^r^ (amplified using the primers JL18-3 and JL18-4) was cloned to the aforementioned plasmid pUC57(Δ*aceA*) (linearized using the primers JL18-1 and JL18-2) using Gibson Assembly. The resulting plasmid pUC57(Δ*aceA*)-P_T5_-*acr1*-*spec*^r^ was used to transform the strain ADP1 Δ*acr1* to obtain the strain W1. To construct the strain ADP1 Acr1, an Acr1-negative strain was first constructed by transforming the wild type ADP1 with the plasmid pUC57(Δ*acr1*) containing the sequences flanking the gene *acr1* in the ADP1 genome (purchased from Genscript, USA). Then, the cassette P_T5_-*acr1*-*spec*^r^ (amplified with the primers JL19-1 and JL19-2) was cloned to the linearized plasmid pIM1463 (linearized with the primers JL19-3 and JL19-4) using USER cloning. The plasmid pIM1463 contains the sequences flanking the prophage region in the ADP1 genome (Murin et al., 2012). The resulting prophage region integration cassette P_T5_-*acr1*-*spec*^r^ was used to transform the Acr1-negative strain to obtain the strain ADP1 Acr1. The strain ADP1 Δ*aceA* ​+ ​iluxAB and W1+iluxAB were constructed by transforming ADP1 Δ*aceA* and W1, respectively, with a previously described cassette containing the genes *luxA* and *luxB* (Santala et al., 2011a). The strain ADP1 Acr1+iluxAB was constructed by transforming the strain ADP1 FAR-neg.+iluxAB with the aforementioned prophage region integration cassette P_T5_-*acr1*-*spec*^r^. For the construction of the strain W2+iluxAB, the plasmid pUC57(Δ*aceA*)- P_T5_-*acr1*-*kan*^r^ was first constructed by cloning the gene *acr1* (amplified from the genome of *A. baylyi* ADP1 using the primers tl17 and sa16) to the plasmid pUC57(Δ*aceA*)- P_T5_-*kan*^r^ by digestion and ligation. The resulting plasmid was used to transform the Acr1-negative strain. The resulting strain was further transformed with the previously described cassette (Lehtinen et al., 2018a) to obtain the strain W2+iluxAB. The cassette contains the genes *luxA* and *luxB* and was designed to knock out the three adjacent genes *poxB* (ACIAD3381), metY (ACIAD3382) and *acr1* (ACIAD3383). The kanamycin resistance marker in the original cassette was replaced with a spectinomycin resistance marker.

### 2.3 Flux balance analysis

Flux balance analysis (FBA) (Orth et al., 2010) was used to predict gene essentiality and calculate the theoretical yields of the conversion of the 6 ​L-amino acids (alanine, asparagine, aspartic acid, glutamic acid, glutamine, and proline) and glucose into WEs by ADP1. A previously described genome-wide *A. baylyi* metabolic network (Durot et al., 2008) was used for the implementation of FBA. To allow simulation of WE accumulation, an exchange reaction of WEs was added in the metabolic network. In all conducted FBAs for essentiality prediction and WE yield calculation, the model was given 1 ​g/h/g CDW of each carbon source as an input (with the default minimal medium components and unlimited oxygen), and the growth reaction and the WE exchange reaction were maximized for respectively.

### 2.4 Characterization of amino acid utilization and fatty aldehyde production

The strains carrying the genes *luxA* and *luxB* were used for the characterization of amino acid utilization or fatty aldehyde production from amino acids/glucose. The studied strains were cultivated in duplicate on 96-well plates (200 ​μl medium/well). The media used is described in the “Strains and media” section. The plates were incubated in Spark multimode microplate reader (Tecan, Switzerland) at 25 ​°C. Double orbital shaking was performed for 5 ​min twice an hour with an amplitude of 6 ​mm and a frequency of 54 ​rpm. Optical density at 600 ​nm (OD_600_) and luminescence signal were measured every hour. The studied strains expressed luciferase LuxAB that reacts with fatty aldehydes and produce luminescence, enabling monitor of fatty aldehyde production.

### 2.5 Cultivation for wax ester production

The cells were first taken from the agar plates containing LB medium and 1% glucose with corresponding antibiotics (if necessary, antibiotics were not added in subsequent cultivations) for pre-cultivation. Pre-cultivations were performed in 5 ​ml medium/14 ​ml cultivation tubes with the same medium as used in the WE production cultivations. WE production cultivations were conducted in 50 ​ml medium/250 ​ml Erlenmeyer flasks at 25 ​°C and 300 ​rpm. The cultivations were carried out in duplicate with a starting OD_600_ of 0.1, using both glucose medium and nitrogen-rich media (media containing casamino acids, yeast extract, and baker’s yeast hydrolysate respectively) as described in “Strains and media” section. For the cultivation with glucose, cells were cultivated for 24 ​h and 48 ​h before harvesting. Considering that WEs might be degraded after the carbon source is depleted, cells were harvested before reaching the stationary phase in nitrogen-rich media which contain 10 ​g/L, 20 ​g/L casamino acids and 50% baker’s yeast hydrolysate. When cultivated with yeast extract, cells were harvested at 5 ​h (before reaching stationary phase) and 24 ​h.

### 2.6 Analytical methods

The baker’s yeast hydrolysate was prepared as described in the “Strains and media” section. Protease was added for the hydrolysis of proteins into amino acids. The concentration of amine groups before and after protease digestion was determined by Ninhydrin Assay. To determine the protein content of the yeast sample, the biomass was heated in 0.5 ​N NaOH at 80–85 ​°C for 30 ​min, allowing the release of protein. Bradford Assay was performed for protein concentration measurement.

WEs were analyzed by the thin layer chromatography (TLC) analysis after small-scale lipid extraction, as described earlier (Lehtinen et al., 2018a). Briefly, proper amounts of cells were harvested from the cultures by centrifugation. WEs were extracted from the cells with methanol-chloroform extraction method. The lower phase was used for TLC analysis, using glass HPTLC Silica Gel 60 F_254_ plate (Merck, USA). The mobile phase was a mixture of hexane, diethyl ether and acetic acid with a ratio of 90:15:1. Jojoba oil was used as the standard of WEs. Visualization was carried out with iodine.

Consumption of glucose was analyzed by high-performance liquid chromatography (HPLC). The analysis was performed with LC-20AC prominence liquid chromatograph (Shimadzu, USA) equipped with RID-10A refractive Index detector. Phenomenex Rezex RHM-monosaccharide H+ (8%) column (Phenomenex, USA) was used and 0.01 ​N sulfuric acid was used as a mobile phase with a pumping rate of 0.6 ​ml/min.

Quantification of WEs was carried out using ^1^H nuclear magnetic resonance (NMR). The cells were harvested from 40 ​ml culture by centrifugation at 30000 *g* for 30 ​min. The harvested cells were freeze-dried with Christ APLHA1-4 LD plus freeze dryer (Germany) for 24 ​h. The extraction of lipids from the freeze-dried cells and analysis with NMR were performed as described previously (Santala et al., 2011a). The chemical shift of 4.05 ​ppm corresponds to the proton signal of α-alkoxy-methylene group of WEs. The integral value of the signal at 4.05 ​ppm was used to calculate the content of WEs. An average molar mass of 506 ​g/mol of WEs was used to calculate the titer, yield, productivity and content (Lehtinen et al., 2018a).

## 3 Results and discussion

### 3.1 Metabolic engineering for redirecting carbon flow from biomass to lipid synthesis

Commercialization of microbial lipid production requires the development of efficient cell catalysts. Previous metabolic engineering strategies have successfully demonstrated optimizing the production organisms, such as by blocking the competing pathways (Lu et al., 2008), increasing the supply of precursors (Lu et al., 2008; Tai and Stephanopoulos, 2013), deregulation of fatty acid synthesis pathway (Lu et al., 2008; Zhang et al., 2012) or balancing the reducing power (Qiao et al., 2017). However, challenges related to the competition between biomass formation and lipid synthesis remain. In addition, the tendency to accumulate lipids in oleaginous species, such as oleaginous yeast, depends on nitrogen limitation due to strict regulation (Garay et al., 2014). Typically, nitrogen limitation coupled with an excess of carbon is used for lipid accumulation. In the strategy, biomass is accumulated in the first stage and lipid accumulation occurs upon nitrogen exhaustion. On one hand, the two-stage process can lower productivity, and nitrogen limitation may not be suitable for cells (over)expressing non-native activities. On the other hand, lipid production from nitrogen-rich substrates, which represent abundant by-products in current industrial scenarios (Li et al., 2018), can be difficult.

WEs have been previously produced by *A. baylyi* ADP1 from various carbon sources, including alkanes (Ishige et al., 2002), glucose (Kannisto et al., 2017; Lehtinen et al., 2018a), acetate and butyrate (Salmela et al., 2018), and aromatic compounds (Salmela et al., 2019). However, WE production from nitrogen-rich substrates has not been described, possibly due to the unfavorable C/N ratio to induce lipid accumulation. We have previously observed WE production during the exponential phase in *A. baylyi* ADP1 (Santala et al., 2018, 2011a), indicating that nutrient limitation is not a strict requirement for lipid accumulation. We hypothesized that proper redirection of carbon flow from biomass formation to lipid synthesis could enable enhanced WE accumulation and improved WE production from both the optimal substrate, namely glucose, and from non-optimal substrates such as nitrogen-rich substrates. To validate the hypothesis, we constructed a strain W1 in which the gene *aceA* was replaced with the gene *acr1* under the control of a strong constitutive promoter, and the native *acr1* was deleted. The gene *acr1* encodes for a fatty acyl-CoA reductase, which reduces fatty acyl-CoAs to fatty aldehydes, the key intermediate for WE synthesis. During the verification, a deletion of nine amino acid residues was found in Acr1 of W1 (Fig. S1). Although the modified version of Acr1 (Acr1∗) was found to be functional in terms of wax ester production, we further evaluated the effect of the deletion on the enzyme activity by specifically monitoring the intracellular fatty aldehyde formation by a previously described sensor (section 2.4). When compared to unmodified Acr1, it was found out that the deletion mutation did not have a negative effect on the production of fatty aldehydes (Fig. S2) in the studied conditions. Thus, the W1 strain with Acr1∗ was used in the later experiments.

The gene *aceA* encodes for isocitrate lyase, catalyzing the first step of the glyoxylate shunt, in which isocitrate is cleaved into succinate and glyoxylate (Fig. 1B). Glyoxylate is subsequently combined with one molecule of acetyl-CoA to form malate. Deletion of *aceA* blocks the glyoxylate shunt, hypothetically increasing the availability of acetyl-CoA for the fatty acid synthesis pathway and energy generation (Fig. 1A). The downstream pathway was up-regulated by *acr1* overexpression for directing the generated acetyl-CoA to WEs (Fig. 1B). It was previously shown that overexpression of *acr1* led to a two-fold improvement in WE production with a titer of 0.45 ​g/L (yield of 0.04 ​g/g glucose) (Lehtinen et al., 2018a). In the previous study, *acr1* was expressed under a lac/T5 -promoter and the highest WE production was obtained with the highest IPTG-concentration studied. Thus, here we decided to use the constitutively expressed T5-promoter. We studied WE production in the engineered strain from both glucose and nitrogen-rich substrates, namely casamino acids, yeast extract and baker’s yeast hydrolysate.

The essentiality of isocitrate lyase AceA to the cells when grown on acetate, glucose and 20 common L-amino acids was predicted using FBA by maximizing for the growth reaction before and after *aceA* deletion. As expected, the gene *aceA* was predicted to be essential when acetate was used as sole carbon source; the theoretical specific growth rate did not change after *aceA* deletion when glucose or amino acids were used as carbon source (Table S1). We further inspected the change of the theoretical maximum yield of WEs from glucose and amino acids when increasing the carbon flux through isocitrate cleavage reaction. The inputs of different carbon sources (glucose and different amino acids) were set to 1 ​g/h/g CDW, and WE accumulation was maximized for. Flux through the isocitrate cleavage reaction in the model decreased the maximum WE yield that was achievable (Table S2). As the yields obtained with basic FBA consider only (genome-scale) stoichiometry but not kinetics or exact thermodynamics, the effect of *aceA* deletion on WE production is not expected to be reflected in the simulation.

### 3.2 Fatty aldehyde and WE production from glucose by engineered ADP1

**Fig. 2:**
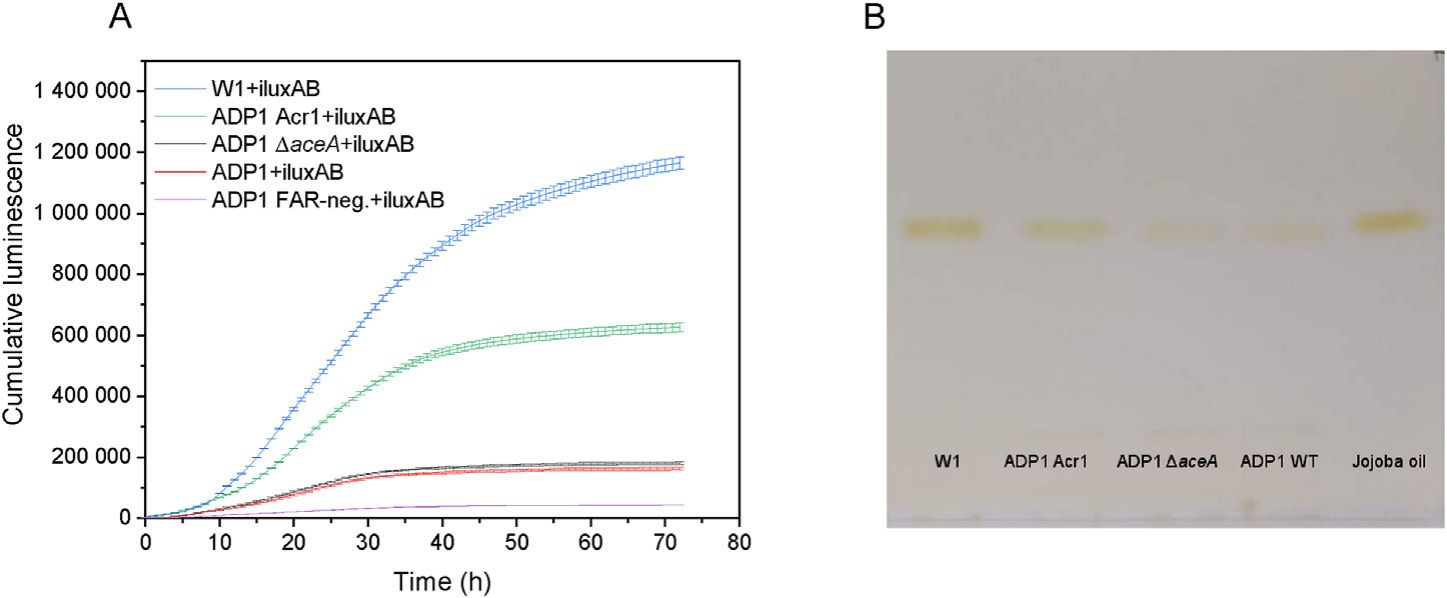
(A) Cumulative luminescence signal generated by W1+iluxAB, ADP1 Acr1+iluxAB, ADP1 Δ*aceA* ​+ ​iluxAB, ADP1+iluxAB and ADP1 FAR-neg.+iluxAB when grown in 200 ​mM glucose. All the strains express bacterial luciferase LuxAB that produces luminescence when reacting with the WE synthesis pathway intermediates, fatty aldehydes, indicating the relative activity of the synthesis pathway. The results represent the mean of two replicates and the error bars represent the standard deviations. (B) Visualization of the produced WEs by TLC (Thin-layer chromatography) analysis. The strains W1, ADP1 Acr1, ADP1 Δ*aceA,* and ADP1 WT were cultivated in 200 ​mM glucose for 48 ​h. For each strain, the same amount of biomass was taken for lipid extraction. Jojoba oil was used as the standard for WEs. Two replicates were analyzed and one representative image is shown.

Fatty aldehydes are specific intermediates in the WE synthesis pathway (Fig. 1), and its production would reflect the carbon flux towards the WE synthesis pathway. Our previously developed bacterial luciferase-based biosensor enables monitoring the intracellular production of fatty aldehydes in real-time during cultivations (Lehtinen et al., 2018a, 2017; Santala et al., 2011a). Bacterial luciferase (LuxAB) catalyzes the oxidation of FMNH_2_ (reduced flavin mononucleotide) and long-chain fatty aldehydes, resulting in the emission of light (Szittner and Meighen, 1990). The biosensor was employed to study the production of fatty aldehydes in the strain W1 (*aceA* deletion and *acr1* overexpression) by the introduction of the gene *luxA* and *luxB*. The resulting strain was designated as W1+iluxAB. For comparison, the following LuxAB-expressing strains were used as controls: ADP1 Acr1+iluxAB (*acr1* overexpression under T5 promoter), ADP1 Δ*aceA* ​+ ​iluxAB (*aceA* deletion), ADP1+iluxAB (wild type with *luxAB*) and ADP1 FAR-neg.+iluxAB (fatty acyl-CoA reductase negative, negative control). These strains were cultivated with 200 ​mM glucose as carbon source and luminescence was measured every hour. All the strains showed prominent growth during the cultivation, detected as changes in OD_600_ (Fig. S3). It has been previously shown that the luminescence signal correlates with the synthesis of intracellular fatty aldehydes (Lehtinen et al., 2018a; Santala et al., 2011a). The signal from ADP1 Δ*aceA* ​+ ​iluxAB was similar or slightly higher than the signal from ADP1 iluxAB (Fig. 2A). By contrast, a significantly higher signal was detected from the *acr1* overexpression strain ADP1 Acr1+iluxAB, indicating increased intracellular fatty aldehyde production. The highest luminescence signal was observed with W1+iluxAB (Fig. 2A), indicating that the overexpression of Acr1 combined with *aceA* knock-out might be beneficial in terms of WE production. To evaluate whether the increased fatty aldehyde production would lead to improved WE synthesis, a 48-h cultivation was performed with the following non LuxAB-expressing strains: W1, ADP1 Acr1, ADP1 Δ*aceA,* and ADP1 WT. After the cultivation, the ODs of the four strains reached 11.9, 11.1, 9.6, and 9.6, respectively. For each strain, the same amount of biomass was taken for lipid extraction to represent WE production at cell level. The extracted lipids were analyzed with TLC. As expected, a correlation between the luminescence signals and the TLC band intensities were observed (Fig. 2A and B). ADP1 Δ*aceA* and ADP1 WT had similar WE contents. A higher WE content was observed with ADP1 Acr1, and W1 produced the highest WE content. Since the highest OD_600_ was also observed with W1, the highest content also led to the highest titer and productivity.

The flux partitioning between the glyoxylate shunt and TCA cycle is generally under regulation (Dolan and Welch, 2018). The glyoxylate shunt in glucose-grown cells is usually repressed for some extensively studied model strains, such as *E.coli* K strains (Dolan and Welch, 2018). However, the partitioning is not an all-or-none switch and its regulation varies in different bacteria; a significant activity of the glyoxylate shunt has been observed in *E.coli* B strain, *Klebsiella pneumoniae* and *Pseudomonas aeruginosa* when grown on glucose (Berger et al., 2014; Bl et al., 2004; Rajput et al., 2013). *A. baylyi* ADP1 is closely related to *P. aeruginosa*, but their regulations in the glyoxylate shunt can also be different. For example, some *Acinetobacter* (including ADP1) genomes lack the gene *aceK* encoding for isocitrate dehydrogenase kinase/phosphatase (Dolan and Welch, 2018), which plays an important role in partitioning the flux between the glyoxylate shunt and the TCA cycle and exists in both *E. coli* and *P. aeruginosa*. It remains to be explored how the flux is partitioned between the glyoxylate shunt and TCA cycle in *A. baylyi* ADP1. If the glyoxylate shunt is used as an anaplerotic route in ADP1 at the studied conditions, *aceA* deletion should lead to increased availability of acetyl-CoA. However, the deletion of *aceA* alone seemed not to have a significant effect on the production of fatty aldehydes/WEs, whereas *aceA* deletion and *acr1* overexpression had a synergistic effect and led to significantly improved production. One possible reason is that despite an increased capacity of acetyl-CoA production, the carbon flux towards the synthesis of fatty aldehydes/WEs was limited by later steps of the pathway, or was restricted by the regulation of the fatty acid synthesis pathway (Janβen and Steinbüchel, 2014). Nevertheless, the carbon flux was not as efficiently directed to WE synthesis in ADP1 Δ*aceA* or ADP1 Acr1 as in W1. It has been reported that in *E. coli* a 100-fold increase in malonyl-CoA levels led to only a 6-fold increase in fatty acid synthesis rate (Davis et al., 2000). In another study, it was shown that increase of the acetyl-CoA pool alone achieved by removing the fumarase did not improve the production of fatty acids in *E. coli*, whereas removal of the fumarase together with overexpression of a downstream enzyme of the fatty acid synthesis pathway had a synergistic effect on improving fatty acid production (Tan et al., 2018). It was suggested that this was due to the increased downstream capacity to channel the excessive acetyl-CoA into fatty acid synthesis.

**Fig. 3:**
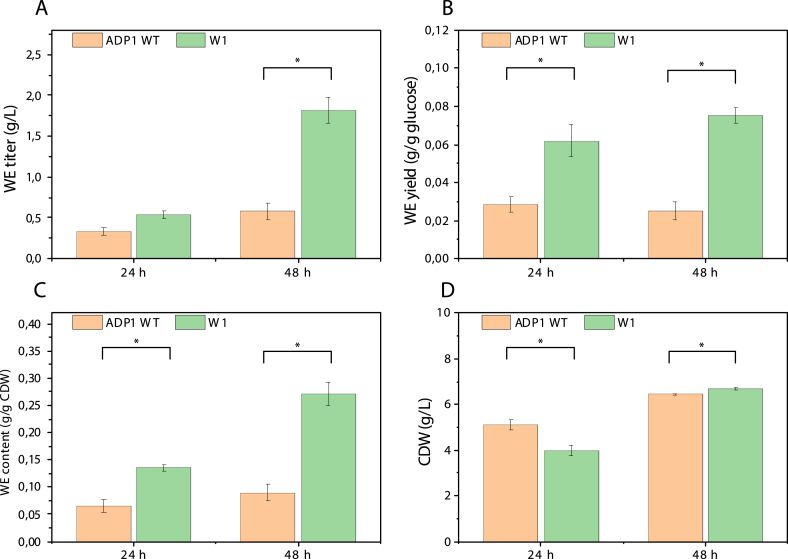
Comparison of (A) WE titer, (B) yield, (C) content and (D) CDW between ADP1 WT and W1 after 24 ​h and 48 ​h of cultivation with 200 ​mM glucose as carbon source. The results represent the mean of two replicates and the error bars represent the standard deviations. ∗*P* ​< ​0.05 (Student’s t-test was implemented for the comparisons between ADP1 WT and the mutant strain: two-tailed, two-sample assuming equal variances).

To quantify the production of WEs by W1, a batch-cultivation was carried out in flasks with 200 ​mM glucose as carbon source. ADP1 WT was used as a control. The two strains were cultivated for 24 ​h and 48 ​h, after which the cells were harvested, and lipids were extracted for WE quantification with NMR. As shown in Fig. 3, significantly improved WE production was observed in W1. After 24 ​h, W1 had a lower cell dry weight (CDW) than ADP1 WT but with a much higher WE yield and content, resulting in a 1.6-fold higher titer. After 48 ​h, W1 had produced a WE titer of 1.82 ​g/L (3.15-fold higher than ADP1 WT) and a yield of 0.075 ​g/g glucose (3-fold higher than ADP1 WT) (Fig. 3A and B), accumulating up to 0.27 ​g/g CDW of WEs (3-fold higher than ADP1 WT) (Fig. 3C). W1 had a slightly higher CDW than ADP1 WT after 48 ​h (Fig. 3D). These results suggest that, when glucose was used as a carbon source, *acr1* overexpression in combination with *aceA* deletion significantly enhanced WE accumulation without reducing cell growth, resulting in improved titer, productivity, and yield. As the glyoxylate shunt is absent in the strain W1, the TCA cycle intermediates that were withdrawn for the synthesis of amino acids or other cell constituents can only be replenished by phosphoenolpyruvate carboxylation since another anaplerotic pathway, pyruvate carboxylation, is not found in the ADP1 metabolic network (Durot et al., 2008).

### 3.3 Characterization of amino acid utilization and fatty aldehyde production from amino acids by engineered ADP1

**Fig. 4:**
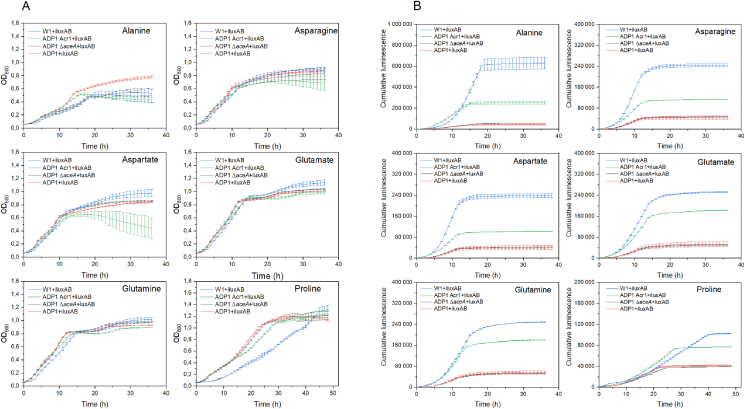
(A) Growth (determined as OD_600_) and (B) cumulative luminescence of W1+iluxAB, ADP1 Acr1+iluxAB, ADP1 Δ*aceA* ​+ ​iluxAB, and ADP1+iluxAB grown on alanine, asparagine, aspartate, glutamate, glutamine, and proline (the 6 amino acids can be used by *A. baylyi* ADP1 as sole carbon source). All the strains express bacterial luciferase LuxAB that produces luminescence when reacting with the WE synthesis pathway intermediates, fatty aldehydes, indicating the relative activity of the synthesis pathway. The results represent the mean of two replicates and the error bars represent the standard deviations.

The obtained results indicate that *acr1* overexpression in combination with *aceA* deletion significantly enhances WE accumulation when glucose is used as a carbon source. The same strategy is expected to benefit WE accumulation when cells are cultivated with nitrogen-rich substrates. Prior to using nitrogen-rich substrates for WE production, we first characterized the utilization of twenty single L-amino acids as sole carbon sources by *A. baylyi* ADP1. The sensor strain ADP1+iluxAB was used for the characterization to obtain information about the activity of the WE synthesis pathway during growth on amino acids. The results showed that ADP1 was able to use six out of the twenty amino acids, namely alanine, asparagine, aspartate, glutamate, glutamine, and proline, as a sole carbon source (Fig. 4A). The ability of ADP1 to use different amino acids as sole carbon source was consistent with the prediction by FBA (Table S1), except for arginine, which could not be used as a sole carbon source by ADP1. The utilization of arginine was predicted by FBA due to the arginine succinyltransferase (AST) pathway, which is present in the ADP1 model. In *E. coli*, the pathway allows arginine utilization as a nitrogen source but not carbon source, putatively due to a lack of promoter activity or inadequate transport during carbon-limited growth (Schneider et al., 1998). It was previously hypothesized that the inability of ADP1 to use arginine as a sole carbon source is due to the same reason (Durot et al., 2008). The six amino acids undergo different catabolic pathways. According to the metabolic network of ADP1 (Durot et al., 2008), L-alanine can be directly converted to pyruvate through transamination by alanine aminotransferase. In an alternative pathway, L-alanine is first converted to D-alanine by alanine racemase, after which the D-alanine is deaminated to form pyruvate by D-alanine dehydrogenase. Pyruvate can be converted to acetyl-CoA by the pyruvate dehydrogenase complex or to phosphoenolpyruvate by phosphoenolpyruvate synthase. Catabolism of the other five amino acids leads to the formation of TCA cycle intermediates: asparagine and proline/glutamine enter the TCA cycle via aspartate and glutamate respectively; aspartate can be further converted to oxaloacetate (through transamination) or fumarate (through deamination) while glutamate is converted to 2-ketoglutarate. Decarboxylation of the TCA cycle intermediates malate and oxaloacetate leads to the formation of pyruvate and phosphoenolpyruvate, which are important for acetyl-CoA formation and gluconeogenesis.

We next cultivated the LuxAB-expressing strains W1+iluxAB, ADP1 Acr1+iluxAB, ADP1 Δ*aceA* ​+ ​iluxAB, and ADP1+iluxAB with 15 ​mM of either alanine, asparagine, aspartate, glutamate, glutamine, or proline. The growth of the different strains on the six amino acids had only small differences (Fig. 4A); ADP1+iluxAB reached higher OD_600_ than other strains on alanine, and W1+iluxAB grew slightly more slowly than other strains on proline. The deletion of *aceA* did not have a negative effect on amino acid utilization. The non-essentiality of isocitrate lyase AceA to the cells, when grown on the six amino acids, was consistent with the prediction by FBA. The luminescence signals generated by ADP1+iluxAB and ADP1 Δ*aceA* ​+ ​iluxAB were similar for all the amino acids, indicating that no significant differences occur in the WE synthesis pathway between the strains in the studied conditions (Fig. 4B). Induction of isocitrate lyase has been observed in *Acinetobacter* spp. in pyruvate medium (Herman and Bell, 1970), indicating that the glyoxylate shunt might be active in alanine medium as alanine catabolism leads to the formation of pyruvate. However, blockage of the shunt only led to a slight increase of the luminescence signal when cells were grown on alanine, and the signal was even slightly lower for ADP1 Δ*aceA* ​+ ​iluxAB when grown on proline. For all the amino acids, a higher cumulative luminescence was generated by ADP1 Acr1+iluxAB, and the highest signal was observed with W1+iluxAB (Fig. 4B). The difference in the cumulative luminescence between W1+iluxAB and ADP1 Acr1+iluxAB was slightly smaller with glutamate, glutamine, and proline than with other amino acids. These results indicate that *acr1* overexpression in combination with *aceA* deletion had a synergistic effect on enhancing the flux towards the WE synthesis pathway when different amino acids were used as sole carbon sources.

### 3.4 WE production from casein amino acids and yeast extract

**Fig. 5:**
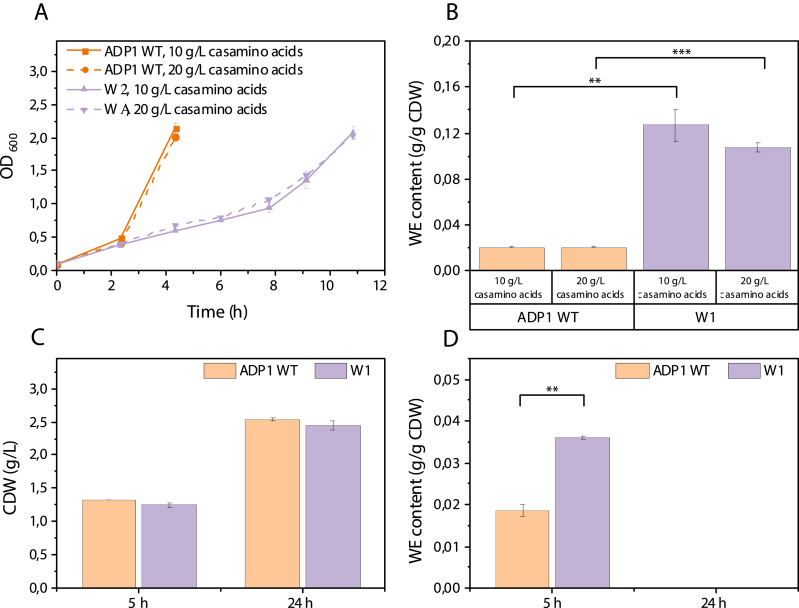
(A) Growth (represented as OD_600_) of the strains W1 and ADP1 WT in 10 and 20 ​g/L casein amino acids. (B) Comparison of WE content between W1 and ADP1 WT in the exponential phase when grown in 10 and 20 ​g/L casein amino acids. Cells of both strains were harvested during exponential phase; ADP1 WT was harvested after 4 ​h, and WI was harvested after 11 ​h. Comparison of (C) CDW and (D) WE content between W1 and ADP1 when cultivated with 20 ​g/L yeast extract. The results represent the mean of two replicates and the error bars represent the standard deviations. ∗∗*P* ​< ​0.01; ∗∗∗*P* ​< ​0.001 (Student’s t-test was implemented for the comparisons between ADP1 WT and W1: two-tailed, two-sample assuming equal variances).

We next studied WE accumulation from casein amino acids (casam) from casein hydrolysate by the strains W1 and ADP1 WT. The strains W1 and ADP1 WT were cultivated in two conditions, with 10 ​g/L and 20 ​g/L casam as carbon source. To compare WE accumulation between the two strains, the cells were harvested when OD_600_ was about 2 ​at which the cells were still in exponential phase, considering that WEs might be rapidly degraded under carbon limiting conditions (Santala et al., 2011a). As shown in Fig. 5A, the concentration of casam did not have a significant influence on the growth of the strains. ADP1 WT grew faster and reached OD_600_ of 2 after 4 ​h while W1 had a slower growth and reached OD_600_ of 2 after 11 ​h. Despite being in nitrogen-rich conditions, WT ADP1 still accumulated a small amount of WEs during the exponential phase, with a content of 0.02 ​g/g CDW in both 10 and 20 ​g/L casam (Fig. 5B). In comparison, W1 accumulated approximately 0.13 and 0.11 ​g/g CDW of WEs in the cultivations with 10 ​g/L and 20 ​g/L casam, respectively (Fig. 5B), suggesting 5–6 folds enhanced accumulation of WEs during exponential phase. Thus, the cellular WE content of W1 from casam was similar to that obtained with ADP1 WT from glucose.

To study the WE accumulation using yeast extract, a cultivation with 20 ​g/L yeast extract as a substrate was performed. The cells were harvested during the exponential phase (5 ​h) and after reaching the stationary phase (24 ​h). Interestingly, W1 and ADP1 WT showed similar growth, and the difference of CDW between the two strains was not apparent when harvested after 5 ​h of cultivation (Fig. 5C). Although W1 had 1.9-fold higher WE content than ADP1 WT (Fig. 5D), the content (0.036 ​g/g CDW) was much lower than those obtained with casam. The difference in cell growth phase may affect the content of WEs, but the main reason for the lower WE content might be attributed to the composition difference between casam and yeast extract. Apart from free amino acids and peptides, yeast extract also contains other nutrients, such as vitamins, macro and micro minerals, which may benefit growth more than lipid accumulation. After 24 ​h, WEs were not detected from either strain likely due to degradation (Fig. 5D). Under the nitrogen-rich condition, carbon source was likely to be the growth-limiting factor during the stationary phase, and the accumulated WEs could be consumed as energy and carbon source. It should be noted that yeast extract was the only carbon source to test the WE production capability solely from yeast extract. It would be beneficial to use small amounts of glucose as a supporting carbon source, if nitrogen-rich substrates were to be used for lipid production. In addition, further engineering, such as blocking β-oxidation by deletion of related genes (Lu et al., 2008; Steen et al., 2010), could be a potential strategy to prevent WE degradation.

Here, we demonstrated a novel metabolic engineering strategy that resulted in significantly improved WE production in nitrogen-rich conditions. However, further engineering is still needed to obtain high WE titer from protein-rich substrates. The *A. baylyi* strain employed in the study can only utilize 6 amino acids as the sole carbon source. For the application point of view, it is important to develop a strain that can utilize more amino acids as carbon sources. Chemical mutagenesis has been shown to be an effective way to improve amino acid utilization (Huo et al., 2011), and the obtained *E. coli* mutant was able to utilize 13 individual amino acids as sole carbon source, compared to 4 for the wild type strain. Moreover, microbes have evolved mechanisms that favor amino acid anabolism (Wernick and Liao, 2013). A driving force for deamination would allow more carbon skeletons to be released from amino acids for product formation, and the strategies for creating the driving force have been well demonstrated in *E. coli* by Huo et al. (Huo et al., 2011). It was shown that deamination could be facilitated by deletion of genes related to the ammonium-assimilation pathway (Huo et al., 2011; Mikami et al., 2017). Furthermore, some amino acids are preferentially degraded by transamination reactions, through which the amino group can be redistributed to glutamate and branched-chain amino acids. These branched-chain amino acids serve as nitrogen reservoirs and cannot be used for chemical production due to the amino group attached to the carbon skeleton. Introduction of exogenous transamination and deamination could allow the release of the carbon skeleton, further draining amino acids that serve as nitrogen reservoirs (Huo et al., 2011).

### 3.5 WE production from baker’s yeast hydrolysate

**Fig. 6:**
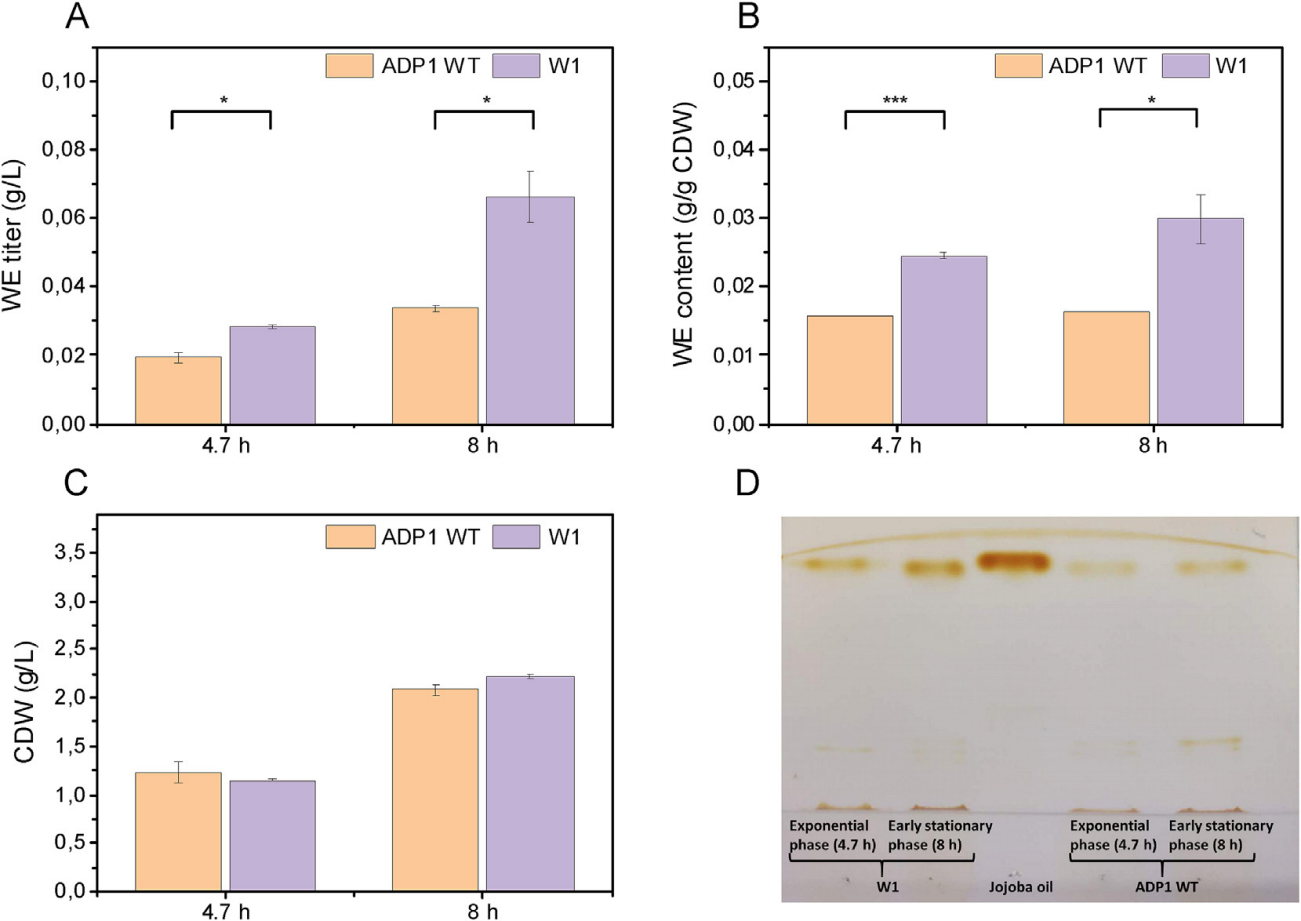
Comparison of (A) WE titer, (B) content and (C) CDW between ADP1 WT and W1 when grown in 50% baker’s yeast hydrolysate. The results represent the mean of two replicates and the error bars represent the standard deviations. (D) WE visualization by TLC. For each strain, the same amount of biomass was taken for lipid extraction. The extracted lipid was analyzed with TLC. Jojoba oil was used as the standard of WEs. ∗*P* ​< ​0.05; ∗∗∗*P* ​< ​0.001 (Student’s t-test was implemented for the comparisons between ADP1 WT and the mutant strain: two-tailed, two-sample assuming equal variances).

Yeast waste represents the second major by-product in the brewery industry and is conventionally sold as inexpensive animal feed (Ferreira et al., 2010). To evaluate the possibility of using yeast biomass for WE production, dry baker’s yeast was used as a representative feedstock. To obtain baker’s yeast hydrolysate that can be used as a substrate, a heat treatment was performed for the yeast biomass to release proteins, followed by digestion with protease to break the peptide bonds of the released proteins. The medium was supplemented with 50% baker’s yeast hydrolysate for cultivation. Cells were harvested during exponential phase (after 4.7 ​h) and early stationary phase (after 8 ​h) where the WE content is likely to be the highest. The CDWs of the strains W1 and ADP1 WT did not differ significantly after 4.7 and 8 ​h of cultivation (Fig. 6C). W1 accumulated 0.025 ​g/g WEs of CDW in the exponential phase and 0.030 ​g/g CDW in the early stationary phase, while the WE content of ADP1 WT did not increase at the end of the cultivation and was maintained at around 0.016 ​g/g CDW (Fig. 6B). The difference of the WE content between the two strains can be also seen through WE visualization (Fig. 6D). The final WE titer of 0.066 ​g/L was obtained with WI, which was 2-fold higher than that obtained with ADP1 WT (Fig. 6A). The results suggest that the protein-rich biomass can be a good feedstock for microbial production; the fermentable hydrolysate can be obtained by a simple pretreatment process.

## 4 Conclusions

In this study, we engineered *A. baylyi* ADP1 to improve the production of WEs by overexpression of *acr1* and deletion of *aceA*. As a result of redirecting the carbon flux, the engineered strain produced 27% WEs of CDW with a titer of 1.8 ​g/L from glucose, representing an approximately 3-fold improvement in production metrics (titer, yield, and productivity) compared to WT. We further demonstrated the possibility to use nitrogen-rich substrates for WE production by *A. baylyi* ADP1. The engineering led to 5–6 fold improvement in WE accumulation from casein amino acids; the amount of WEs produced by the engineered strain was similar to that produced by ADP1 WT from glucose. The study represents the starting point for establishing efficient high-value lipid production from non-optimal substrates such as protein-rich waste streams. We also anticipate that this approach could provide insight for improving the production of other oleochemicals.

## Author contribution

JL, SS, and VS designed the study. JL carried out the microbiological and molecular work. PL and JL conducted flux balance analysis. EE and JL conducted substrate and WE analysis. All authors participated in writing the manuscript. All authors read and approved the final manuscript.

## CRediT authorship contribution statement

**Jin Luo:** Conceptualization, Methodology, Software, Validation, Formal analysis, Investigation, Writing - original draft, Writing - review & editing. **Elena Efimova:** Methodology, Formal analysis, Investigation, Writing - original draft. **Pauli Losoi:** Methodology, Software, Writing - original draft. **Ville Santala:** Conceptualization, Methodology, Writing - original draft, Writing - review & editing, Supervision, Funding acquisition. **Suvi Santala:** Conceptualization, Methodology, Writing - original draft, Writing - review & editing, Supervision, Funding acquisition.

## Declaration of competing interest

The authors declare that they have no known competing financial interests or personal relationships that could have appeared to influence the work reported in this paper.

## References

[bib1] Berger A., Dohnt K., Tielen P., Jahn D., Becker J., Wittmann C. (2014). Robustness and plasticity of metabolic pathway flux among uropathogenic isolates of Pseudomonas aeruginosa. PloS One.

[bib2] Bl E.B., Jm E.K., Phue J., Shiloach J. (2004). Transcription levels of key metabolic genes are the cause for different glucose utilization pathways in Acetyl-P Acetyl-AMP. J. Biotechnol..

[bib3] Breuer G., Lamers P.P., Martens D.E., Draaisma R.B., Wijffels R.H. (2012). The impact of nitrogen starvation on the dynamics of triacylglycerol accumulation in nine microalgae strains. Bioresour. Technol..

[bib4] Davis M.S., Solbiati J., Cronan J.E. (2000). Overproduction of acetyl-CoA carboxylase activity increases the rate of fatty acid biosynthesis in Escherichia coli. J. Biol. Chem..

[bib5] de Berardinis V., Vallenet D., Castelli V., Besnard M., Pinet A., Cruaud C., Samair S., Lechaplais C., Gyapay G., Richez C., Durot M., Kreimeyer A., Le Fèvre F., Schächter V., Pezo V., Döring V., Scarpelli C., Médigue C., Cohen G.N., Marlière P., Salanoubat M., Weissenbach J. (2008). A complete collection of single-gene deletion mutants of Acinetobacter baylyi ADP1. Mol. Syst. Biol..

[bib6] Dolan S.K., Welch M. (2018). The glyoxylate shunt, 60 Years on. Annu. Rev. Microbiol..

[bib7] Doong S.J., Gupta A., Prather K.L.J. (2018). Layered dynamic regulation for improving metabolic pathway productivity in Escherichia coli. Proc. Natl. Acad. Sci..

[bib8] Durot M., Le Fèvre F., de Berardinis V., Kreimeyer A., Vallenet D., Combe C., Smidtas S., Salanoubat M., Weissenbach J., Schachter V. (2008). Iterative reconstruction of a global metabolic model of Acinetobacter baylyi ADP1 using high-throughput growth phenotype and gene essentiality data. BMC Syst. Biol..

[bib9] Ferreira I.M.P.L.V.O., Pinho O., Vieira E., Tavarela J.G. (2010). Brewer’s Saccharomyces yeast biomass: characteristics and potential applications. Trends Food Sci. Technol..

[bib10] Fixter L.M., Nagi M.N., Mccormack J.G., Fewson C.A. (1986). Structure, distribution and function of wax esters in acinetobacter calcoaceticus. Microbiology.

[bib11] Garay L.A., Boundy-Mills K.L., German J.B. (2014). Accumulation of high-value lipids in single-cell microorganisms: a mechanistic approach and future perspectives. J. Agric. Food Chem..

[bib12] Herman N.J., Bell E.J. (1970). Metabolic control in Acinetobacter sp. I. Effect of C 4 versus C 2 and C 3 substrates on isocitrate lyase synthesis. Can. J. Microbiol..

[bib13] Huo Y.-X., Cho K.M., Rivera J.G.L., Monte E., Shen C.R., Yan Y., Liao J.C. (2011). Conversion of proteins into biofuels by engineering nitrogen flux. Nat. Biotechnol..

[bib14] Ishige T., Tani A., Takabe K., Kawasaki K., Sakai Y., Kato N. (2002). Wax ester production from n-alkanes by acinetobacter sp. strain M-1: ultrastructure of cellular inclusions and role of acyl coenzyme A reductase. Appl. Environ. Microbiol..

[bib15] Janßen H.J., Steinbüchel A. (2014). Fatty acid synthesis in Escherichia coli and its applications towards the production of fatty acid based biofuels. Biotechnol. Biofuels.

[bib16] Kalscheuer R., Steinbüchel A. (2003). A novel bifunctional wax ester synthase/acyl-CoA:diacylglycerol acyltransferase mediates wax ester and triacylglycerol biosynthesis in acinetobacter calcoaceticus ADP1. J. Biol. Chem..

[bib17] Kannisto M., Efimova E., Karp M., Santala V. (2017). Growth and wax ester production of an Acinetobacter baylyi ADP1 mutant deficient in exopolysaccharide capsule synthesis. J. Ind. Microbiol. Biotechnol..

[bib18] Kornberg H. (1966). The role and control of the glyoxylate cycle in Escherichia coli. Biochem. J..

[bib19] Lehtinen T., Efimova E., Santala S., Santala V. (2018). Improved fatty aldehyde and wax ester production by overexpression of fatty acyl-CoA reductases. Microb. Cell Factories.

[bib20] Lehtinen T., Santala V., Santala S. (2017). Twin-layer biosensor for real-time monitoring of alkane metabolism. FEMS Microbiol. Lett..

[bib21] Lehtinen T., Virtanen H., Santala S., Santala V. (2018). Production of alkanes from CO2 by engineered bacteria. Biotechnol. Biofuels.

[bib22] Li S.Y., Ng I.S., Chen P.T., Chiang C.J., Chao Y.P. (2018). Biorefining of protein waste for production of sustainable fuels and chemicals. Biotechnol. Biofuels.

[bib23] Lu X., Vora H., Khosla C. (2008). Overproduction of free fatty acids in E. coli: implications for biodiesel production. Metab. Eng..

[bib24] Luo J., Lehtinen T., Efimova E., Santala V., Santala S. (2019). Synthetic metabolic pathway for the production of 1-alkenes from lignin-derived molecules. Microb. Cell Factories.

[bib25] Metzgar D. (2004). Acinetobacter sp. ADP1: an ideal model organism for genetic analysis and genome engineering. Nucleic Acids Res..

[bib26] Mikami Y., Yoneda H., Tatsukami Y., Aoki W., Ueda M. (2017). Ammonia production from amino acid-based biomass-like sources by engineered Escherichia coli. Amb. Express.

[bib27] Murin C.D., Segal K., Bryksin A., Matsumura I. (2012). Expression vectors for acinetobacter baylyi ADP1. Appl. Environ. Microbiol..

[bib28] Orth J.D., Thiele I., Palsson B.Ø. (2010). What is flux balance analysis?. Nat. Biotechnol..

[bib29] Peralta-Yahya P.P., Zhang F., del Cardayre S.B., Keasling J.D. (2012). Microbial engineering for the production of advanced biofuels. Nature.

[bib30] Pfleger B.F., Gossing M., Nielsen J. (2015). Metabolic engineering strategies for microbial synthesis of oleochemicals. Metab. Eng..

[bib31] Qiao K., Wasylenko T.M., Zhou K., Xu P., Stephanopoulos G. (2017). Lipid production in Yarrowia lipolytica is maximized by engineering cytosolic redox metabolism. Nat. Biotechnol..

[bib32] Ragauskas A.J. (2006). The path forward for biofuels and biomaterials. Science.

[bib33] Rajput M.S., Naresh Kumar G., Rajkumar S. (2013). Repression of oxalic acid-mediated mineral phosphate solubilization in rhizospheric isolates of Klebsiella pneumoniae by succinate. Arch. Microbiol..

[bib34] Reiser S., Somerville C. (1997). Isolation of mutants of Acinetobacter calcoaceticus deficient in wax ester synthesis and complementation of one mutation with a gene encoding a fatty acyl coenzyme A reductase. J. Bacteriol..

[bib35] Rontani J.-F. (2010). Production of wax esters by bacteria. Handbook of Hydrocarbon and Lipid Microbiology.

[bib36] Salmela M., Lehtinen T., Efimova E., Santala S., Mangayil R. (2018). Metabolic pairing of aerobic and anaerobic production in a one-pot batch cultivation. Biotechnol. Biofuels.

[bib37] Salmela M., Lehtinen T., Efimova E., Santala S., Santala V. (2019). Alkane and wax ester production from lignin related aromatic compounds. Biotechnol. Bioeng. bit.

[bib38] Sánchez M., Avhad M.R., Marchetti J.M., Martínez M., Aracil J. (2016). Jojoba oil: a state of the art review and future prospects. Energy Convers. Manag..

[bib39] Santala S., Efimova E., Karp M., Santala V. (2011). Real-Time monitoring of intracellular wax ester metabolism. Microb. Cell Factories.

[bib40] Santala S., Efimova E., Kivinen V., Larjo A., Aho T., Karp M., Santala V. (2011). Improved triacylglycerol production in acinetobacter baylyi ADP1 by metabolic engineering. Microb. Cell Factories.

[bib41] Santala S., Efimova E., Koskinen P., Karp M.T., Santala V. (2014). Rewiring the wax ester production pathway of acinetobacter baylyi ADP1. ACS Synth. Biol..

[bib42] Santala S., Efimova E., Santala V. (2018). Dynamic decoupling of biomass and wax ester biosynthesis in Acinetobacter baylyi by an autonomously regulated switch. Metab. Eng. Commun..

[bib43] Schneider B.L., Kiupakis A.K., Reitzer L.J. (1998). Arginine catabolism and the arginine succinyltransferase pathway in Escherichia coli. J. Bacteriol..

[bib44] Singh S., Bhadani A., Singh B. (2007). Synthesis of wax esters from α-olefins. Ind. Eng. Chem. Res..

[bib45] Soma Y., Tsuruno K., Wada M., Yokota A., Hanai T. (2014). Metabolic flux redirection from a central metabolic pathway toward a synthetic pathway using a metabolic toggle switch. Metab. Eng..

[bib46] Steen E.J., Kang Y., Bokinsky G., Hu Z., Schirmer A., McClure A., Del Cardayre S.B., Keasling J.D. (2010). Microbial production of fatty-acid-derived fuels and chemicals from plant biomass. Nature.

[bib47] Suárez G.A., Renda B.A., Dasgupta A., Barrick J.E. (2017). Reduced mutation rate and increased transformability of transposon-free acinetobacter baylyi ADP1-ISx. Appl. Environ. Microbiol..

[bib48] Szittner R., Meighen E. (1990). Nucleotide sequence, expression, and properties of luciferase coded by lux genes from a terrestrial bacterium. J. Biol. Chem..

[bib49] Tai M., Stephanopoulos G. (2013). Engineering the push and pull of lipid biosynthesis in oleaginous yeast Yarrowia lipolytica for biofuel production. Metab. Eng..

[bib50] Tan Z., Yoon J.M., Chowdhury A., Burdick K., Jarboe L.R., Maranas C.D., Shanks J.V. (2018). Engineering of E. coli inherent fatty acid biosynthesis capacity to increase octanoic acid production. Biotechnol. Biofuels.

[bib51] Tuck C.O., Perez E., Horvath I.T., Sheldon R.A., Poliakoff M. (2012). Valorization of biomass: deriving more value from waste. Science (80-.

[bib52] Tumen-Velasquez M., Johnson C.W., Ahmed A., Dominick G., Fulk E.M., Khanna P., Lee S.A., Schmidt A.L., Linger J.G., Eiteman M.A., Beckham G.T., Neidle E.L. (2018). Accelerating pathway evolution by increasing the gene dosage of chromosomal segments. Proc. Natl. Acad. Sci..

[bib53] Wältermann M., Steinbüchel A. (2005). Neutral lipid bodies in prokaryotes: recent insights into structure, formation, and relationship to eukaryotic lipid depots. J. Bacteriol..

[bib54] Wernick D.G., Liao J.C. (2013). Protein-based biorefining: metabolic engineering for production of chemicals and fuel with regeneration of nitrogen fertilizers. Appl. Microbiol. Biotechnol..

[bib55] Zhang F., Ouellet M., Batth T.S., Adams P.D., Petzold C.J., Mukhopadhyay A., Keasling J.D. (2012). Enhancing fatty acid production by the expression of the regulatory transcription factor FadR. Metab. Eng..

