# Supplementary material for: Wax ester production in nitrogen-rich conditions by metabolically engineered Acinetobacter baylyi ADP1
Source: Metab Eng Commun. 2020 Apr 25;10:e00128. doi: 10.1016/j.mec.2020.e00128 (PMC7251950; doi:10.1016/j.mec.2020.e00128)
Supplement: Multimedia component 1 [file mmc1.doc]

**Supplementary material**

**Table S1.** Theoretical specific growth rate (1/h) of the strain ADP1 WT and ADP1 Δ*aceA* on different carbon sources. Flux balance analysis (FBA) was used for calculating the specific growth rate. The same genome-wide *A. baylyi* metabolic network was used for both strains in FBA, except that the gene *aceA* was deleted from the network for ADP1 Δ*aceA*. In all conducted FBAs, 1 g/h/g CDW of each carbon source was set as an input (with the default minimal medium components and unlimited oxygen), and the growth reaction was maximized for.

|  | **ADP1 WT** | **ADP1 Δ*aceA*** |
| --- | --- | --- |
| **Acetate** | 0.413 | 0 |
| **Ala** | 0.465 | 0.46 |
| **Arg** | 0.416 | 0.416 |
| **Asn** | 0.33 | 0.33 |
| **Asp** | 0.321 | 0.321 |
| **Cys** | 0 | 0 |
| **Glucose** | 0.453 | 0.453 |
| **Gln** | 0.451 | 0.451 |
| **Glu** | 0.438 | 0.438 |
| **Gly** | 0 | 0 |
| **His** | 0 | 0 |
| **Ile** | 0 | 0 |
| **Leu** | 0 | 0 |
| **Lys** | 0 | 0 |
| **Met** | 0 | 0 |
| **Phe** | 0 | 0 |
| **Pro** | 0.629 | 0.629 |
| **Ser** | 0 | 0 |
| **Thr** | 0 | 0 |
| **Trp** | 0 | 0 |
| **Tyr** | 0 | 0 |
| **Val** | 0 | 0 |

**Table S2.** Theoretical yield of WEs (expressed as g WEs/g carbon source) from different carbon sources by *A. baylyi* ADP1 (table on the left) and the theoretical yield when the flux through isocitrate cleavage reaction was increased from 0 mmol/h/g CDW to 5 mmol/h/g CDW (table on the right). FBA was used for calculating the theoretical yield. In all conducted FBAs, 1 g/h/g CDW of each carbon source was set as an input (with the default minimal medium components and unlimited oxygen), and the exchange reaction of WEs was maximized for.

|  | Flux through isocitrate cleavage reaction (mmol/h/g CDW) | | | | | | |
| --- | --- | --- | --- | --- | --- | --- | --- |
|  | **0** | **0.1** | **0.25** | **0.5** | **1** | **2.5** | **5** |
| **Ala** | 0.305 | 0.305 | 0.305 | 0.304 | 0.303 | 0.258 | 0.184 |
| **Arg** | 0.17 | 0.167 | 0.163 | 0.155 | 0.14 | 0.096 | 0.022 |
| **Asn** | 0.21 | 0.21 | 0.21 | 0.209 | 0.195 | 0.15 | 0.076 |
| **Asp** | 0.204 | 0.204 | 0.204 | 0.203 | 0.193 | 0.149 | 0.074 |
| **Cys** | 0 | 0 | 0 | 0 | 0 | 0 | 0 |
| **Glucose** | 0.281 | 0.281 | 0.28 | 0.28 | 0.279 | 0.255 | 0.181 |
| **Gln** | 0.203 | 0.2 | 0.195 | 0.188 | 0.173 | 0.129 | 0.055 |
| **Glu** | 0.201 | 0.198 | 0.194 | 0.187 | 0.172 | 0.127 | 0.053 |
| **Gly** | 0 | 0 | 0 | 0 | 0 | 0 | 0 |
| **His** | 0 | 0 | 0 | 0 | 0 | 0 | 0 |
| **Ile** | 0 | 0 | 0 | 0 | 0 | 0 | 0 |
| **Leu** | 0 | 0 | 0 | 0 | 0 | 0 | 0 |
| **Lys** | 0 | 0 | 0 | 0 | 0 | 0 | 0 |
| **Met** | 0 | 0 | 0 | 0 | 0 | 0 | 0 |
| **Phe** | 0 | 0 | 0 | 0 | 0 | 0 | 0 |
| **Pro** | 0.257 | 0.254 | 0.25 | 0.243 | 0.228 | 0.183 | 0.109 |
| **Ser** | 0 | 0 | 0 | 0 | 0 | 0 | 0 |
| **Thr** | 0 | 0 | 0 | 0 | 0 | 0 | 0 |
| **Trp** | 0 | 0 | 0 | 0 | 0 | 0 | 0 |
| **Tyr** | 0 | 0 | 0 | 0 | 0 | 0 | 0 |
| **Val** | 0 | 0 | 0 | 0 | 0 | 0 | 0 |

| **Ala** | 0.305 |
| --- | --- |
| **Arg** | 0.17 |
| **Asn** | 0.21 |
| **Asp** | 0.204 |
| **Cys** | 0 |
| **Glucose** | 0.281 |
| **Gln** | 0.203 |
| **Glu** | 0.201 |
| **Gly** | 0 |
| **His** | 0 |
| **Ile** | 0 |
| **Leu** | 0 |
| **Lys** | 0 |
| **Met** | 0 |
| **Phe** | 0 |
| **Pro** | 0.257 |
| **Ser** | 0 |
| **Thr** | 0 |
| **Trp** | 0 |
| **Tyr** | 0 |
| **Val** | 0 |

**Table S3.** List of primers used in the study

| Name | Description | Oligo sequence (5-3’) |
| --- | --- | --- |
| tl17 | XbaI, amplification of the gene *acr1* (ACIAD3383) | TGGAATTCGCGGCCGCTTCTAGAGAAAGAGGAGAAATACTAGATGATATCAATCAGGGAAAAACGCG |
| sa16 | XhoI, amplification of the gene *acr1* (ACIAD3383) | CTTCTTCTCGAGTTATTACCAGTGTTCGCCTGG |
| tl45 | XhoI, amplification of spectinomycin resistance marker | GTAGCGCTCGAGGCAGAAAGGAGAAGCTTACTAGC |
| tl46 | PstI, amplification of spectinomycin resistance marker | ATCTTGCTGCAGCTCGGCTTGAACGAATTGTTAGAC |
| JL18-1 | Linearization of the plasmid pUC57 containing the flanking sequences of the gene *aceA* for Gibson Assembly | GGCGTATGGTTTAAAAAAC |
| JL18-2 | Linearization of the plasmid pUC57 containing the flanking sequences of the gene *aceA* for Gibson Assembly | GATATATTCCCTTTTAGGATTTC |
| JL18-3 | Amplification of the cassette P_T5_-*acr1*-*spec*^r^ for Gibson Assembly | ATCCTAAAAGGGAATATATCCAATTGGCTGGCATCCCTAAC |
| JL18-4 | Amplification of the cassette P_T5_-*acr1*-*spec*^r^ for Gibson Assembly | GGTTTTTTAAACCATACGCCCCTAGGGCTTAATGCGCC |
| 1084 V1 | Confirmation of the deletion of the gene *aceA* (ACIAD1084) | TTTTTCTATCATTCATTTTTAAGTC |
| 1084 V2 | Confirmation of the deletion of the gene *aceA* (ACIAD1084) | CTCAACATGATATGCACACTGC |
| JL19-1 | Amplification of the cassette the cassette P_T5_-*acr1*-*spec*^r^ for USER cloning | AAGCAAUTGGCTGGCATCCCTAACATATCC |
| JL19-2 | Amplification of the cassette the cassette P_T5_-*acr1*-*spec*^r^ for USER cloning | ACTCCCUAGGGCTTAATGCGCCGCT |
| JL19-3 | Linearization of the plasmid pIM1463 for USER cloning | AGGGAGUGTTCATATTGACCTCGCTTAGTG |
| JL19-4 | Linearization of the plasmid pIM1463 for USER cloning | ATTGCTUGAGTGAGGTCCGTTCCTATGC |


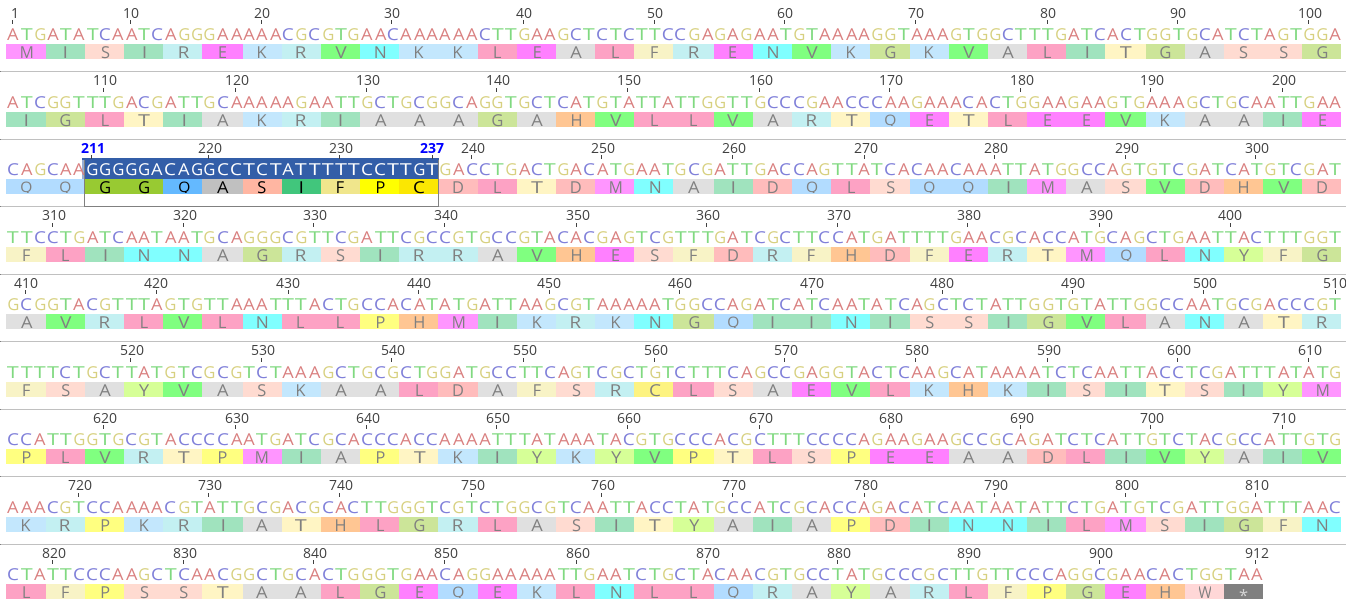


**Figure S1.** Sequence of the gene *acr1* (ACIAD3383) of *A.baylyi* ADP1. The deletion (CDS 211-237) in the *acr1* of the strain W1 is highlighted.

As a mutation that results in the deletion of nine amino acid residues was found in the gene *acr1* (responsible for fatty aldehyde generation from fatty acyl-CoA) of the strain W1, the effect of the mutation on the production of fatty aldehyde, the intermediate for WE synthesis, was studied. Our previously developed luciferase (LuxAB)-based biosensor enables monitoring intracellular fatty aldehyde production in real-time (Lehtinen et al., 2017, 2018; Santala et al., 2011). The sensor was employed for the study by introduction of the genes *luxA* and *luxB* into the cells. The two resulting strains were designated as W1+iluxAB and W2+iluxAB. In both strains, the gene *aceA* was knocked out and the gene *acr1* was under the control of a T5 promoter, but the *acr1* in W1+iluxAB contains the found mutation while the one in W2+iluxAB does not have the mutation. Both strains were cultivated in 200 mM glucose. The cumulative luminescence signal detected from W1+iluxAB was very similar to that from W2+iluxAB, indicating comparable productions of fatty aldehyde by the two strains. Thus, it was concluded that the mutation had no effect on the enzymatic activity of Acr1.

**Figure S2.** (A) Change of OD_600_ over time for the strains W1+iluxAB, W2+iluxAB, and ADP1 FAR-neg.+iluxAB. (B) Cumulative luminescence generated by the studied strains. All the strains express luciferase LuxAB that produces luminescence when reacting with the fatty aldehyde. The cells were cultivated on 96-well plate in 200 mM glucose. The strain ADP1 FAR-neg.+iluxAB was used as the negative control as the strain does not contain the fatty acyl-CoA reductase and therefore does not produce fatty aldehydes. . The results represent the mean of two replicates and the error bars represent the standard deviations.

**Figure S3.** Change of OD_600_ over time for the strains W1+iluxAB, ADP1 Acr1+iluxAB, ADP1 Δ*aceA*+iluxAB, ADP1+iluxAB and ADP1 FAR-neg.+iluxAB when grown in 200 mM glucose. The cells were cultivated on 96-well plate. The results represent the mean of two replicates and the error bars represent the standard deviations.


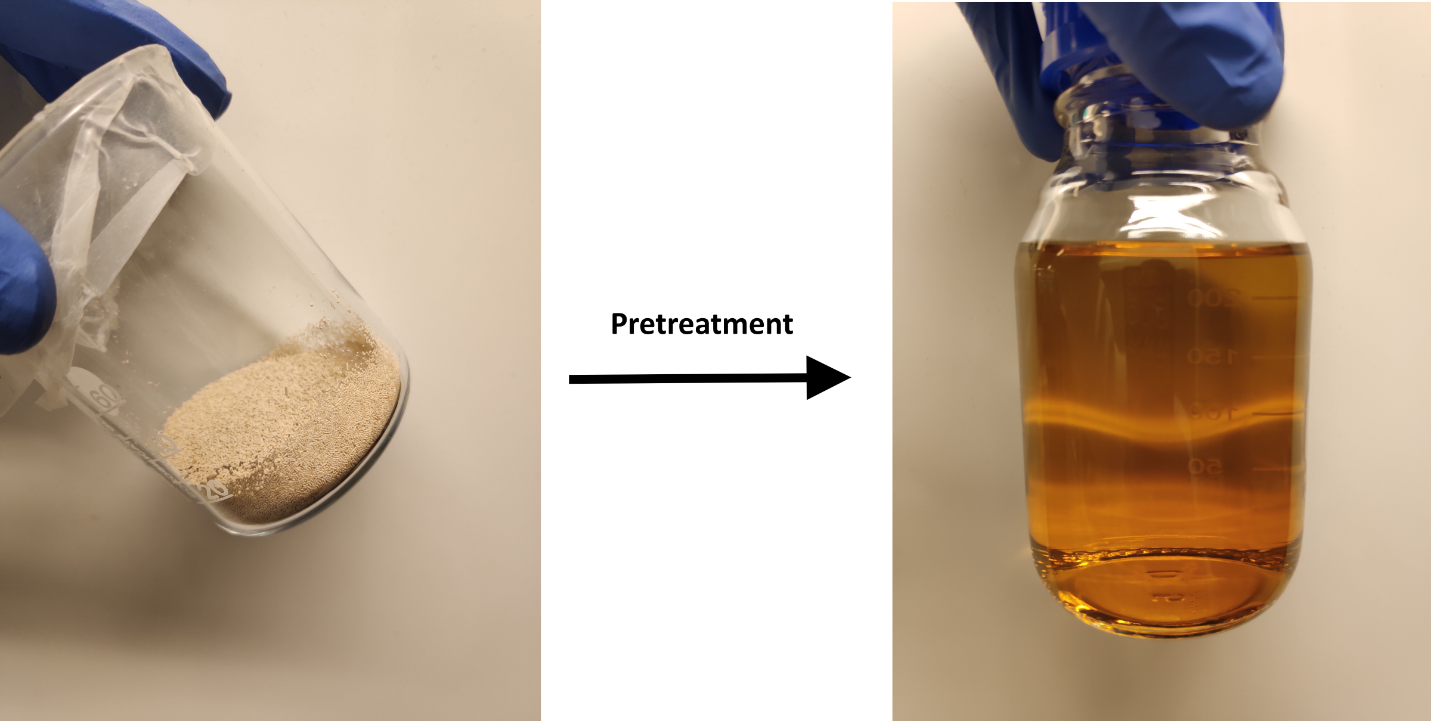


**Figure S4.** Dry baker yeast (on the left) and its hydrolysate stock solution (on the right). 126 g/L of the biomass was heated in water at 80-85 degree for 18 min, allowing the release of 44582 μM free amine groups measured by Ninhydrin test. The biomass was then digested with 1-2 g/L of protease at 60 degree overnight, further increasing the amount of amine group to 72450 μM. The hydrolysate was centrifuged, and the supernatant was filtered for use as a substrate.

**References**

Lehtinen, T., Efimova, E., Santala, S., & Santala, V. (2018). Improved fatty aldehyde and wax ester production by overexpression of fatty acyl-CoA reductases. *Microbial Cell Factories*, *17*(1), 19. https://doi.org/10.1186/s12934-018-0869-z

Lehtinen, T., Santala, V., & Santala, S. (2017). Twin-layer biosensor for real-time monitoring of alkane metabolism. *FEMS Microbiology Letters*, *364*(6), 1–7. https://doi.org/10.1093/femsle/fnx053

Santala, S., Efimova, E., Karp, M., & Santala, V. (2011). Real-Time monitoring of intracellular wax ester metabolism. *Microbial Cell Factories*, *10*(1), 75. https://doi.org/10.1186/1475-2859-10-75
